# Association Between Inflammatory Potential of the Diet and Ulcerative Colitis: A Case-Control Study

**DOI:** 10.3389/fnut.2020.602090

**Published:** 2021-02-10

**Authors:** Zeinab Khademi, Parvane Saneei, Ammar Hassanzadeh-Keshteli, Hamed Daghaghzadeh, Hamid Tavakkoli, Peyman Adibi, Ahmad Esmaillzadeh

**Affiliations:** ^1^Students' Scientific Research Center, Tehran University of Medical Sciences, Tehran, Iran; ^2^Department of Community Nutrition, School of Nutritional Sciences and Dietetics, Tehran University of Medical Sciences, Tehran, Iran; ^3^Food Security Research Center, Isfahan University of Medical Sciences, Isfahan, Iran; ^4^Department of Community Nutrition, School of Nutrition and Food Science, Isfahan University of Medical Sciences, Isfahan, Iran; ^5^Department of Medicine, University of Alberta, Edmonton, AB, Canada; ^6^Integrative Functional Gastroenterology Research Center, Isfahan University of Medical Sciences, Isfahan, Iran; ^7^Obesity and Eating Habits Research Center, Endocrinology and Metabolism Molecular -Cellular Sciences Institute, Tehran University of Medical Sciences, Tehran, Iran

**Keywords:** ulcerative colitis, inflammatory bowel disease, inflammatory potential of the diet, dietary pattern, case-control study

## Abstract

**Background/Aim:** Despite the inflammatory nature of inflammatory bowel disease (IBD), limited data are available on the association of inflammatory potential of the diet and risk of ulcerative colitis (UC). We aimed to investigate the association of inflammatory potential of the diet (IPD) score and odds of UC in a case-control study.

**Methods:** Patients with UC were enrolled from Iranian IBD registry, whose disease was confirmed by a gastroenterologist. Controls were selected randomly from the Study of the Epidemiology of Psycho Alimentary Health and Nutrition (SEPAHAN) study, a large population-based study on more than 8,000 apparently healthy individuals. Dietary intakes of 28 food items obtained from a validated dish-based food frequency questionnaire (FFQ), were used to compute IPD score.

**Results:** This case-control study was carried out among 109 cases and 218 randomly chosen controls. Mean age of cases and controls was 39.5 ± 10.0 and 41.5 ± 11.8 y, respectively. Totally, 52% of study participants were female and 48% were male. After controlling for age, sex, and body mass index (BMI), we found that the patients with UC were more likely to be in the highest quartile of IPD score compared with controls (OR: 2.83; 95% CI: 1.41–5.69, *P*-trend < 0.001). This association strengthened after additional adjustment for education, smoking, medical history, and physical activity (OR: 3.48; 95% CI: 1.32–9.10, *P*-trend = 0.003). When we took dietary habits into account, the association was slightly attenuated (OR: 3.33; 95% CI: 1.20–9.20, *P*-trend = 0.005).

**Conclusions:** We found that adherence to a pro-inflammatory diet was positively associated with greater odds of UC. Further studies are required to confirm these findings.

## Introduction

Ulcerative colitis (UC), as an inflammatory bowel disease (IBD), is a multifactorial disorder characterized by chronic, relapsing, and progressive inflammatory condition (1, 2). It starts from the rectum and may involve the entire colon (3). In UC, inflammation is typically superficial and limited to the mucosa and submucosa (3, 4). According to a national survey in Iran, the prevalence of UC has been estimated to be 40.67 per 100,000 subjects (5).

Although the exact etiology is yet unknown, it seems that UC may be the result of an inappropriate and continuing inflammatory response to altered gut microbiota in a genetically susceptible host (2, 4). However, the role of environmental factors including smoking, stress, and diet cannot be ignored (2, 6, 7). Concerning the inflammatory nature of UC, dietary components can play a role in UC pathogenesis through modulation of inflammation (8, 9). For instance, adherence to Western dietary pattern, which is low in food items with anti-inflammatory and anti-oxidant proprieties, was linked to an increased risk of UC (10), while consumption of Mediterranean dietary pattern, rich in anti-inflammatory agents including fruits, vegetables, fish, and nuts, decreased the risk of UC (11).

One of the emerging indices to determine the inflammatory potential of the whole diet is dietary inflammatory index (DII). DII is a literature-derived population-based index, developed and construct validated (12–14) by Shivappa et al. (15, 16). This index has widely been used to examine the link between inflammatory potential of the diet and odds of many chronic conditions, even gastrointestinal disorders. For instance, a greater empirically derived food-based dietary inflammatory index (FDII) was associated with an increased risk of irritable bowel syndrome (IBS) in an Iranian study (17). Adherence to a pro-inflammatory diet was also related to the risk of reflux esophagitis (18). With regards to UC, we are aware of only one report in which 62 patients with UC and 124 hospitalized controls were enrolled. In that case-control study, consumption of a more pro-inflammatory diet was associated with 1.5 times greater odds of UC (19). However, a limitation of that study was the use of hospital-based controls, in whom dietary habits may differ from the general population. Additionally, some potential confounders were not considered.

Limited studies that investigated the association between diet and UC risk are conducted in western countries. Dietary patterns in western countries differ from those in Middle Eastern countries. High intakes of refined carbohydrates and saturated fatty acids and low intake of fiber are common in the Middle East. Such dietary intakes might be associated with increased risk of UC. Accordingly, assessing the relationship between diet and IBD is particularly relevant in these countries. Therefore, we conducted a case-control study that aimed to evaluate the association between inflammatory potential of the diet and risk of UC in Iran.

## Materials and Methods

### Study Participants

This case-control study was done between 2015 and 2019 in Isfahan, Iran. Cases were patients diagnosed with UC by a gastroenterologist that had been registered in Iranian IBD registry. Before enrolment, the study design and aims were explained to all registered patients (*n* = 140) during an educational class on lifestyle, then patients were requested to participate in this study. Out of these 140 patients, 109 people agreed to participate. It is noteworthy that there was no diversity concerning general characteristics including age, physical activity, and residence area between those who agreed to participate, and those who did not. Controls were randomly selected from the Study of the Epidemiology of Psycho-Alimentary Health and Nutrition (SEPAHAN) project, a large population-based study on more than 8,000 apparently healthy individuals, conducted in the same region. Detailed information about SEPAHAN project can be found elsewhere (20). Before selection of controls, all individuals with gastrointestinal disorders (including Crohn's disease, ulcerative colitis, irritable bowel syndrome, functional dyspepsia, gastro-esophageal reflux disorder) were excluded from the SEPAHAN dataset. Finally, two age- (±2 y) and sex-matched controls for each case were randomly chosen.

### Assessment of Dietary Intakes

A validated self-administrated dish-based Food Frequency Questionnaire (DS-FFQ), that included 106 food items, was used to determine participants' usual dietary intakes. Further information on the design, food list, and validity of this questionnaire has been reported before (21). This questionnaire included five domains of foods and dishes (mixed dishes, potatoes and grain-based foods, dairy products, fruits and vegetables, miscellaneous foods and beverages). We asked each subject to determine his/her usual intakes of these food items in the previous year. They were able to choose one choice from nine multiple-choice options. These choices ranged from “never or less than once a month” to “12 or more times per day.” Subjects' reported intakes were then converted to grams per day, for doing that we used household measures. To calculate nutrient intakes for each person in the study, we applied the US Department of Agriculture nutrient database. However, some parts of this database were modified for Iranian local foods based on Iranian food composition table. Earlier studies on a subgroup of 200 participants indicated that this questionnaire works well in estimating long-term dietary intakes of people (21).

### Construction of Inflammatory Potential of the Diet Score

The DSFFQ-derived data were used to calculate the IPD scores for all subjects. In the current study, we used the method developed by Shivappa et al. (15) to construct IPD score. The development (15) and construct validation of the DII has been described elsewhere (12, 13). Shivappa et al. (15) identified 45 specific foods or nutrients, according to published literature, that were linked with several inflammatory biomarkers: Interleukin-1β (IL-1β), Interleukin-6 (IL-6), Tumor Necrosis Factor-α (TNF-α), C-reactive protein (CRP), Interleukin-4 (IL-4), and Interleukin-10 (IL-10). Moreover, they gave each food or nutrient a specific inflammatory effect score based on its positive or negative association with inflammatory biomarkers in previous publications. If a food or nutrient was positively associated with pro-inflammatory biomarkers, the score of +1 was given to that food parameter. In case of a negative association, the food parameter was given the score of −1, and in case of null association, the score of 0 was given. They also provided world mean and standard deviations for all these 45 food items using data from 11 different countries around the world. Considering lack of consumption of some of these food items in Iranian food culture and having some missing food components (like polyphenols) in Irannian nutrient database, we included the following 28 food items (instead of 45) to calculate the IPD score: energy, carbohydrate, fat, protein, cholesterol, saturated fat, vitamin B12, and iron (pro-inflammatory items) and mono-unsaturated fatty acids (MUFA), polyunsaturated fatty acids (PUFA), fiber, vitamin B6, folic acid, niacin, riboflavin, thiamin, vitamin A, vitamin C, vitamin D, vitamin E, β-carotene, caffeine, pepper, onion, green/black tea, zinc, selenium, and magnesium (anti-inflammatory items). In present study, we calculate z-score for each food parameter by subtracting the “standard global mean” from participants' reported intake for each item and dividing this value by “global standard deviation,” which we took from the paper of Shivappa et al. (15). To reduce the skewness, all z-scores were then converted to percentiles, as earlier studies did (15). Then, we multiplied the percentile scores by the inflammatory effect score of each food parameter derived from the study of Shivappa et al. (15). Finally, we summed up all foods' IPD scores to construct the final IPD score for each participant. A greater IPD score (more positive) represents a more pro-inflammatory diet. Previous studies in Iran has shown the validity of this modified DII index against circulating levels of inflammatory biomarkers (22).

### Assessment of UC

Patients with UC were identified by an experienced gastroenterologist according to international criteria (23) including physical, colonoscopic, and histological examinations. To confirm the diagnosis, we reviewed medical records.

### Assessment of Other Variables

Using a self-administered demographic and medical questionnaire, subjects were able to report required information including age, sex (male/female), marital status (single/married), education (high school graduated or below/ academic education), smoking status (yes/no), family size, homeownership (owner/ non-owner), and medical history including existence of hyperlipidemia, hypertension, gallstone, Crohn's disease, and diabetes. Data on dietary habits including meal regularity (never or occasionally/often or always), fluid consumption during meals (<3 glasses/≥3 glasses), chewing efficiency (not a lot/ a lot), fried foods intake (<4 per week/≥4 per week), and fatty meals intake (non-fatty meal/fatty meal) were collected through a pretested dietary habit questionnaire. To examine physical activity of study participants, General Practice Physical Activity Questionnaire (GPPAQ) was applied. Based on the guideline of this questionnaire, we classified study subjects as having no activity, having activity as <3 h per week, 3–5 h per week, 5–7 h per week, and ≥7 h per week. Required information about anthropometric variables was collected through the use of a self-administered questionnaire. Body mass index (BMI) was calculated as weight divided by height squared. Our previous study had shown that self-administered questionnaire of anthropometric measures provides valid information compared with actual measured values (24).

### Statistical Analysis

Required sample size for this study was calculated based on prior evidence indicating that approximately 60% of Iranian adults are following non-healthy dietary patterns (25). According to previous publications, we assumed that consumption of unhealthy dietary patterns would double the risk of IBD (19). Therefore, considering type I error of 5%, the study power of 80%, and 2 controls per case, we needed at least 101 cases and 202 controls for the current study. To categorize study participants, we defined quartile cut-offs points of IPD score in the control group: Q_1_: <−1.70; Q_2_: between −1.70 and −0.41; Q_3_: between −0.41 and 0.97; Q_4_: >0.97. Comparisons between cases and controls were performed by applying student's *t*-test for quantitative variables and chi-square for categorical variables. Significant differences across categories of IPD score were examined using one-way analysis of variance (ANOVA). However, chi-square test was applied to assess the distribution of participants in terms of categorical variables across quartiles of IPD score. We applied analysis of covariance (ANCOVA) to obtain energy, sex, and age-adjusted dietary intakes of subjects across categories of IPD score. Binary logistic regression was used to find the association of IPD score with UC, in crude and multivariable-adjusted models. Potential confounders were selected according to previous studies, in which intended variable was found to be associated with UC (26, 27). In the first model, BMI (continuous), age (continuous), and sex (male/female) were controlled for. Then, in the second model, we additionally adjusted for smoking (yes/no), having diabetes (yes/no), education (high school graduated or below/ academic education), and physical activity (no activity/<3 h per week/3–5 h per week/5–7 h per week/≥7 h per week). In the last model, regular meal consumption (never or occasionally/often or always), fluid consumption during meals (<3 glasses/≥3 glasses), chewing efficiency (not a lot/a lot), fried foods intake (<4 per week/≥4 per week), and consumption of fatty meals (non-fatty meal/fatty meal) were also adjusted for. In all analyses, the first quartile of IPD score was considered as the reference category. In the logistic regression models, we treated quartiles of IPD score as an ordinal variable to determine P for trends. SPSS software version 19 was used to carry out all statistical analyses. *P*-values were considered statistically significant at < 0.05.

## Results

### Characteristics of UC Cases and Controls

In the present study, 327 people (109 cases and 218 controls) were included. Mean age of cases and controls was 39.5 ± 10.0 and 41.5 ± 11.8 y, respectively. Totally, 52% of study participants were female and 48% were male. Participants with UC were less likely to be physically active and university graduates. No significant differences in mean age and BMI were observed between cases and controls. There was also no significant difference in the distribution of subjects when considering them in terms of sex, smoking status, marital status, and history of diabetes. Comparing participants across quartiles of IPD score, we failed to find any significant difference in mean age and BMI as well as sex, marital status, smoking status, history of diabetes, education, and physical activity (Table 1).

**Table 1:** Characteristics of patients with ulcerative colitis (UC) and controls across quartiles of inflammatory potential of the diet (IPD) score.

	**Ulcerative colitis**					**Quartiles of IPD score**								
	**Yes (*****n*** **=** **109)**		**No (*****n*** **=** **218)**			**Q1 (*****n*** **=** **73)**		**Q2 (*****n*** **=** **69)**		**Q3 (*****n*** **=** **79)**		**Q4 (*****n*** **=** **106)**		
	**Mean**	**SD**	**Mean**	**SD**	***P*^†^**	**Mean**	**SD**	**Mean**	**SD**	**Mean**	**SD**	**Mean**	**SD**	***P*^†^**
Age, y	39.5	10.0	41.5	11.8	0.12	40.9	11.1	39.3	10.4	40.2	9.9	40.1	11.0	0.85
BMI, kg/m^2^	25.2	3.48	25.3	3.85	0.72	25.3	3.45	25.7	4.12	25.0	3.39	25.1	3.93	0.70
Sex (Females), %	52		52		0.94	56		46		51		54		0.59
Marital status (Married), %	78		80		0.19	81		81		77		81		0.54
Smoking status (Current smoker), %	6		4		0.46	5		10		4		2		0.17
History of diabetes, %	4		2		0.46	2		3		4		3		0.83
Education (University graduate), %	38		54		0.007	54		50		50		43		0.49
Family size (>4 people), %	6		12		0.054	14		15		8		7		0.21
Home ownership (owner), %	80		59		0.09	67		60		70		65		0.80
Physical activity, %					0.02									0.36
No activity	14		5			10		4		7		8		
<3 h per week	36		29			29		27		29		37		
3–5 h per week	21		23			19		22		24		27		
5–7 h per week	12		11			21		14		10		4		
≥7 h per week	17		32			21		33		30		24		

† *Obtained from ANOVA or chi-squared test, where appropriate*.

### Dietary Habits of UC Cases and Controls

No significant difference was observed between cases and controls in terms of dietary habits including regular meal pattern, chewing efficiency, fluid consumption during meals, fried foods intake, and fatty meals intake. This was also the case when we compared the distribution of subjects in terms of above-mentioned variables across quartiles of IPD score (Table 2).

**Table 2:** Distribution of patients with ulcerative colitis (UC) and controls in terms of dietary habits across quartiles of inflammatory potential of the diet (IPD) score.

	**Ulcerative colitis**			**Quartiles of IPD score**				
	**Yes (*n* = 109)**	**No (*n* = 218)**	***P*^†^**	**Q1 (*n* = 73)**	**Q2 (*n* = 69)**	**Q3 (*n* = 79)**	**Q4 (*n* = 106)**	***P*^†^**
Regular meal pattern (Often or always), %	64	64	0.99	64	61	75	57	0.10
Chewing sufficiency (A lot), %	10	17	0.09	20	19	10	11	0.16
Fluid consumption (≥3 glasses), %	2	5	0.21	4	5	4	3	0.95
Consumption of fried foods (≥ 4 per week), %	12	16	0.30	18	18	9	15	0.36
Consumption of fatty meals (Fatty meal), %	1	2	0.39	4	2	1	2	0.42

† *Obtained from chi-squared test*.

### Dietary Intakes of UC Cases and Controls

Cases reported higher intakes of energy, PUFA, vitamin A, vitamin C, and vitamin B12 and lower intakes of protein, MUFA, vitamin B1, vitamin B2, vitamin B3, iron, zinc, magnesium, β-carotene, folate and, dietary fiber compared to controls. Participants in the highest quartile of IPD score had lower energy, vitamin E, vitamin C, vitamin B6, riboflavin, niacin, β-carotene, folate, dietary fiber, MUFA, PUFA, Fe, Mg, Zn, and, Se as well as higher vitamin B12 and PUFA intake compared with those in lowest quartile (Table 3). In addition, patients with UC had lower intakes of fruits and refined grains than controls, whereas there were no significant differences in consumption of whole-grains, white meats, red and processed meats, fish, vegetables dairy products, nuts and legumes, green/black tea and coffee. Higher IPD score was associated with lower intakes of red and processed meat, and green/black tea and coffee, and higher intake of refined grains (Table 4).

**Table 3:** Dietary intakes of selected nutrients for patients with ulcerative colitis (UC) and controls across quartiles of inflammatory potential of the diet (IPD) score.

	**Ulcerative colitis**					**Quartiles of IPD score**								
	**Yes (*****n*** **=** **109)**		**No (*****n*** **=** **218)**			**Q1 (*****n*** **=** **73)**		**Q2 (*****n*** **=** **69)**		**Q3 (*****n*** **=** **79)**		**Q4 (*****n*** **=** **106)**		
	**Mean**	**SE**	**Mean**	**SE**	***P*^†^**	**Mean**	**SE**	**Mean**	**SE**	**Mean**	**SE**	**Mean**	**SE**	***P*^†^**
Energy (kcal/d)	3,014	101	2,328	69	<0.001	3,530	101	2,825	104	2,382	99	1,825	83	<0.001
**Nutrients**														
Carbohydrates (%)	41.8	26.25	54.63	25.80	0.73	34.45	34.50	45.02	30.38	53.90	30.30	71.23	33.78	0.38
Proteins (%)	11.81	0.07	16.32	7.01	0.005	11.01	9.42	13.60	8.30	15.44	8.28	19.50	9.25	0.053
Total fats (%)	32.54	22.10	40.20	21.65	0.07	28.30	29.13	33.13	25.61	39.67	25.63	50.79	28.51	0.25
Dietary fiber (g/d)	18	0.67	24	0.45	<0.001	30	0.74	25	0.67	20	0.64	16	0.60	<0.001
MUFA (g/d)	29	1.02	40	0.68	<0.001	45	1.37	40	1.24	34	1.18	30	1.10	<0.001
PUFA (g/d)	43	1.08	31	0.72	<0.001	33	1.59	32	1.45	36	1.37	38	1.28	0.02
SFA (g/d)	25	0.61	24	0.41	0.29	25	0.80	24	0.73	25	0.69	24	0.64	0.41
Vitamin B12 (mcg/d)	7.33	0.39	3.41	0.26	<0.001	2.30	0.55	4.06	0.50	4.95	0.47	6.42	0.44	<0.001
Vitamin B6 (mg/d)	1.63	0.04	2.10	0.03	<0.001	2.61	0.05	2.21	0.04	1.82	0.04	1.44	0.04	0.001
Folate (mcg/d)	266	9.14	337	6.13	<0.001	440	9.30	342	8.44	298	8.0	224	7.4	<0.001
Niacin (mg/d)	22	0.49	27	0.33	<0.001	27	0.69	27	0.63	24	0.60	23	0.56	<0.001
Riboflavin (mg/d)	1.74	0.05	1.99	0.34	<0.001	2.2	0.06	2.03	0.05	1.95	0.05	1.67	0.05	<0.001
Thiamine (mg/d)	1.83	0.05	1.97	0.03	<0.001	1.84	0.07	2.07	0.07	1.96	0.06	1.89	0.06	0.09
Vitamin A (RE/d)	934	36	579	24	<0.001	828	52	629	47	672	45	657	42	0.02
Vitamin C (mg/d)	162	8.18	111	5.49	<0.001	167	10.9	130	9.92	124	9.42	101	8.81	<0.001
Vitamin D (mcg/d)	0.86	0.08	1.02	0.05	0.13	1.23	0.11	0.95	0.10	0.89	0.09	0.88	0.09	0.11
Vitamin E (mg/d)	13.2	0.64	22.4	0.43	<0.001	27.5	0.82	22.7	0.75	17.4	0.71	13.4	0.66	<0.001
β-carotene (mcg/d)	293	186	3764	37	<0.001	5722	238	3432	216	2330	205	331	192	<0.001
Fe (mg/d)	15.83	0.33	18.83	0.22	<0.001	19.15	0.46	19.26	0.41	17.57	0.39	16.41	0.37	<0.001
Mg (mg/d)	236	7.3	346	4.91	<0.001	416	8.52	357	7.72	294	7.33	224.4	6.86	<0.001
Zn (mg/d)	10	0.23	12	0.15	<0.001	13	0.29	12	0.26	11	0.25	10	0.23	<0.001

† *All values were adjusted for age, sex and energy, except for dietary energy intake, which was only adjusted for age and sex using ANCOVA*.

**Table 4:** Dietary intakes of selected food groups for patients with ulcerative colitis (UC) and controls across quartiles of inflammatory potential of the diet (IPD) score.

	**Ulcerative colitis**					**Quartiles of IPD score**								
	**Yes (*****n*** **=** **109)**		**No (*****n*** **=** **218)**			**Q1 (*****n*** **=** **73)**		**Q2 (*****n*** **=** **69)**		**Q3 (*****n*** **=** **79)**		**Q4 (*****n*** **=** **106)**		
	**Mean**	**SE**	**Mean**	**SE**	***P*^†^**	**Mean**	**SE**	**Mean**	**SE**	**Mean**	**SE**	**Mean**	**SE**	***P*^†^**
**FOOD GROUPS**														
Refined grains (g/d)	267	17	354	12	<0.001	265	23	322	21	350	20	357	19	0.03
Whole-grains (g/d)	38	7.98	48	55	0.28	53	10	51	9.42	35	8.95	42	8.39	0.52
White meats (g/d)	56	4.20	53	2.82	0.53	58	5.54	53	5.02	52	4.77	54	4.46	0.83
Red and processed meats (g/d)	90	4.44	80	2.98	0.07	96	5.80	86	5.26	74	4.99	76	4.67	0.05
Fish (g/d)	21	5.26	17	3.53	0.58	10	6.93	22	6.29	15	5.97	24	5.58	0.40
Fruits (g/d)	256	19	266	13	<0.001	284	23	244	24	275	22	253	19	0.57
Vegetables (g/d)	197	13	206	8.7	0.60	311	16	216	2	193	13	127	13	<0.001
Dairy products (g/d)	248	28	366	18	0.002	342	36	334	33	374	31	268	30	0.08
Legumes and nuts (g/d)	60	4.10	56	2.73	0.42	71	5.32	52	4.82	55	4.58	53	4.28	0.03
Green/black tea and coffee (g/d)	319	29	377	19	0.09	512	36	349	33	331	32	280	29	<0.001

† *All values were adjusted for age, sex and energy using ANCOVA*.

### Association of IPD With UC

After controlling for age, sex, and BMI, we found that participants in highest quartile of IPD score had 183% increased chance of UC compared with those in the lowest quartile (OR: 2.83; 95% CI: 1.41–5.69, *P*-trend < 0.001). More adjustment for education, smoking, medical history, and physical activity strengthened this association (OR: 3.48; 95% CI: 1.32–9.10, *P*-trend = 0.003). After we took dietary habits into account, the association did not change much (OR: 3.33; 95% CI: 1.20–9.20, *P*-trend = 0.005) (Table 5).

**Table 5:** Crude and multivariable-adjusted ORs and 95% CIs for UC across quartiles of inflammatory potential of the diet (IPD) score.

	**Quartiles of IPD score**										
	**Q1 (*n* = 73)**	**Q2 (*****n*** **=** **69)**		**P**	**Q3 (*****n*** **=** **79)**		**P**	**Q4 (*****n*** **=** **106)**		**P**	
	**OR**	**OR**	**95 % CI**		**OR**	**95 % CI**		**OR**	**95 % CI**		***P*-trend**
**ULCERATIVE COLITIS**											
Crude	1.00	0.67	0.30, 1.49	0.32	1.24	0.61, 2.52	1.24	2.73	1.43, 5.22	0.002	<0.001
Model 1^†^	1.00	0.52	0.21, 1.31	0.16	1.16	0.53, 2.51	0.70	2.83	1.41, 5.69	0.003	<0.001
Model 2^‡^	1.00	0.54	0.16, 1.81	0.34	0.95	0.34, 2.69	0.99	3.48	1.32, 9.10	0.01	0.003
Model 3^§^	1.00	0.49	0.14, 1.72	0.28	0.87	0.30, 2.56	0.87	3.33	1.20, 9.20	0.02	0.005

† *Model 1: Adjusted for age, sex, and BMI*.

‡ *Model 2: Additionally, adjusted for education, smoking, medical history (diabetes), and physical activity*.

§ *Model 3: Further adjusted for regular meal pattern, chewing sufficiency, fluid consumption during a meal, fried food intake, and fatty food intake*.

## Discussion

We found that patients with UC were more likely to consume pro-inflammatory diets compared with controls. This association remained significant after taking potential confounders into account. To the best of our knowledge, this is among the first studies in which the link between inflammatory potential of the diet and UC was assessed.

UC prevalence has rapidly increased in developing countries over recent decades. It causes an enormous cost on the healthcare system and has been shown to negatively affect general health, mental health, and social function of patients (26). Chronic inflammation is an underlying physiological process that has been associated with numerous gastrointestinal disorders including IBS (17), reflux esophagitis (18), and UC (19). In fact, there are several lines of evidence linking inflammation to UC. For instance, consumption of fruits and vegetables, known as anti-inflammatory food parameters (28), was linked to decreased risk of UC (29). While intake of pro-inflammatory foods such as red and processed meats and refined carbohydrates was associated with elevated risk of UC (30–32).

We found that adherence to a pro-inflammatory diet was associated with greater odds of UC. It has been shown that higher diet quality was associated with a lower chance of UC. In a cohort study, higher Healthy Eating Index-2015 (HEI-2015) score was associated with 66% reduced risk of UC (33). This was also reported about dietary TAC score; such that higher dietary total antioxidant capacity (TAC) score was associated with lower odds of UC in a case-control study (34). Only one previous study has examined the relationship between literature-derived nutrient-based DII and UC. Our findings are similar to this hospital-based case-control study, in which consuming a pro-inflammatory diet was associated with 1.5 times increased risk of UC (19). However, a recent prospective cohort study investigating the relation between empirical dietary inflammatory pattern (EDIP) score and IBD incidence, found no significant association between a pro-inflammatory dietary pattern with UC risk (35). The difference in studies' design might help to explain discrepant findings. Prospective studies, especially in developing countries are needed to confirm these findings.

The potential mechanisms through which might a pro-inflammatory diet contributes to UC pathogenesis are yet to be determined. Chronic inflammation is an underlying physiological process associated with inflammatory bowel disease, UC. Certain dietary components may induce inflammation by altering inflammatory gene expression (36, 37). Results from a recent publication showed that a pro-inflammatory diet is associated with greater proinflammatory gene expression among non-obese individuals (38). Considering the role of inflammatory mediators including cytokines in the regulation of intestinal immune responses and intestinal mucosal barrier homeostasis (39), cytokines play a critical role in causing the chronic inflammatory condition in IBD (40). Therefore, consumption of a pro-inflammatory diet can contribute to UC pathogenesis through increasing serum levels of inflammatory cytokines. Moreover, there is growing evidence showing that gut microbiota is associated with a lot of immune and inflammatory disorders. Gut microbiota can influence intestinal immunity by promoting the development and maintenance of the mucosal immune system, protecting against pathogen invasion, and maintaining gastrointestinal tract barrier integrity (41). Abnormalities of the gut microbiome are common in different intestinal conditions, including IBD. In fact, an altered microbiota has been reported in IBD (10, 39). Diet can affect the gut microbiota (42, 43), as a recent publication revealed a correlation between intakes of energy and micronutrients such as vitamin A, vitamin D and, vitamin C, all contributed to IPD score, with fecal and mucosal communities in UC patients (44). Therefore, a pro-inflammatory diet might increase risk of UC through its influence on gut flora composition.

Our study has several strengths. Being among the first studies in the field, using a validated FFQ to assess subjects' dietary intake and statistical adjustment for several potential covariates are among these strengths. However, there are some limitations as well. Findings from case-control study do not allow conferring causality. Therefore, prospective cohort studies would be required to confirm these findings. Recall and selection bias are inherent in case-control studies, which might affect our findings. Although we used a validated FFQ for dietary assessment, there is still the possibility of measurement errors and misclassification of study participants in terms of exposure. Despite controlling for several potential covariates, the effect of residual confounding cannot be excluded. There was a difference in educational status of cases and controls. This has occurred because cases were selected from IBD registry, while controls were chosen from SEPAHAN project dataset, which was conducted among university employees. This difference might lead to a difference in the household income between cases and controls, which can in turn affect the findings. Additionally, the possibility of changing dietary intake in UC patients as a result of their condition cannot be ignored. We did not collect any information about the duration of disease in UC patients, therefore we could not analyze our data accordingly. In the present study, unlike original DII method, we computed IPD score using data on only 28 nutrient or food items. In total, we did not consider 17 items (saffron, garlic, rosemary, turmeric, ginger, oregano, n-3 fatty acid, trans fatty acid, n-6 fatty acids, flavonols, isoflavones, flavan-3-ol, flavonones, flavones, eugenol, anthocyanidins, and alcohol) in our IPD score calculation due to lacking their information in our dataset. Given that most missing items in our dataset were of anti-inflammatory potential, therefore, studies that include all these items in their DII score calculation might reach a different conclusion than the one we found. Other studies were done in Iran, including the one that has been done on UC patients, have used only 27 foods or nutrients to calculate DII. Finally, in the current study, we had no data on inflammatory biomarkers to examine the validity of IPD; however, previous studies in Iran have shown good correlations between DII and serum concentrations of inflammatory biomarkers, indicating a reasonable validity of this index to predict inflammation (45, 46).

## Conclusion

As a conclusion, this case-control study indicated that consuming a diet with high IPD score might be positively associated with UC. Considering the limitations of case-control studies as well as the nutrient-based index we used in the current analysis which cannot capture interactions between nutrients, future studies are required to examine this association. In particular, developing a new index based on all available publications on the association between foods and inflammation is necessary. Designing prospective studies to investigate this association can also shed light on this link in the future.

## Data Availability Statement

The raw data supporting the conclusions of this article will be made available by the authors, without undue reservation.

## Ethics Statement

The studies involving human participants were reviewed and approved by Ethical Committee of the Tehran University of Medical Sciences, Tehran, Iran (IR.TUMS.VCR.REC.1398.497). The patients/participants provided their written informed consent to participate in this study.

## Author Contributions

ZK and PS contributed in conception, design, search, statistical analyses, data interpretation, and manuscript drafting. AH-K and HD contributed in design and data interpretation. HT and PA contributed in conception, design, statistical analyses, data interpretation, and manuscript drafting. AE supervised the study. All authors approved the final manuscript for submission. All authors contributed to the article and approved the submitted version.

## Conflict of Interest

The authors declare that the research was conducted in the absence of any commercial or financial relationships that could be construed as a potential conflict of interest.
